# Facial Baroparesis Mimicking Stroke

**DOI:** 10.5811/cpcem.2018.1.36488

**Published:** 2018-03-14

**Authors:** Diann M. Krywko, D. Tyler Clare, Mohamad Orabi

**Affiliations:** * Medical University of South Carolina, Department of Emergency Medicine, Charleston, South Carolina; † Medical University of South Carolina, Department of Neurology, Charleston, South Carolina

## Abstract

We report a case of a 55-year-old male who experienced unilateral facial muscle paralysis upon ascent to altitude on a commercial airline flight, with resolution of symptoms shortly after descent. The etiology was determined to be facial nerve barotrauma, or facial baroparesis, which is a known but rarely reported complication of scuba diving, with even fewer cases reported related to aviation. The history and proposed pathogenesis of this unique disease process are described.

## INTRODUCTION

Facial baroparesis is a seventh cranial nerve palsy caused by transient hypoxemia of the facial nerve secondary to increased pressure in the middle ear cavity. It has classically been reported in divers, with isolated cases reported in the aviation literature.^1^,^2^,^3^,^4^ The facial nerve travels through the tympanic segment of the facial canal. It is in this segment of its complicated anatomical course that the nerve is thought to be affected by increased pressure. This temporary, ischemic neuropraxia may be relieved by equalizing the pressure in the middle ear, through the nasopharynx via the eustachian tube. We report a case of baroparesis involving a previously healthy 55-year-old male who experienced a transient facial nerve palsy while traveling on commercial aircraft.

## CASE REPORT

A 55-year-old Caucasian male flying between New York City and Miami noticed an increased sensation of pressure in his ears upon ascent, which he was unable to equalize using typical techniques (Valsalva, yawning, chewing gum). At maximum elevation, he experienced a tingling sensation on the left side of his tongue, left-sided facial numbness, dysarthria and a generalized headache. He noticed left-sided facial drooping when looking at his reflection in a mirror. The flight attendant was notified and a mid-air emergency was called. Two orthopedic physician passengers responded and evaluated the patient. Their exam noted left upper and lower facial droop along with dysarthria. Vital signs including blood pressure were within normal limits. Forty-five minutes after symptom onset, an emergency landing was initiated. Upon descent, the patient reported that the pressure sensation started to decrease in his left ear, and suddenly his left ear “popped,” leading to near-complete resolution of symptoms. Repeat exam noted improvement of facial asymmetry. The patient was transported by ambulance to the emergency department (ED) for further evaluation.

In the ED he reported a history of nasal congestion and cough for three weeks prior to the flight. His social history was negative for tobacco or drug use, with social alcohol use reported. Current medications included an unknown antibiotic and Flonase. His physical exam showed the following vital signs: blood pressure 152/88 millimeters of mercury, heart rate 70 beats per minute, temperature 36.9º Celsius, respiratory rate 14 breaths per minute, oxygen saturation 99% on room air. Examination of his head, eyes, ears, nose and throat was unremarkable. Neurologic examination revealed an alert and oriented male in no distress, normal orientation, attention and language. Cranial nerves II–XII were intact, though there was a question of slight left upper-lip asymmetry as described by the emergency physician. His motor, sensation, coordination, and reflexes were all normal. The National Institutes of Health Stroke Scale was zero.

Secondary to the possible asymmetry, the stroke team was activated by the ED. Imaging was obtained including a non-contrasted computed axial tomography (CT) of his head, a CT angiogram of the head and neck, and magnetic resonance imaging with and without contrast of his head and neck. All imaging was normal. Laboratory results showed white blood cell count 10.9 x 10^3^ per microliter, hemoglobin 15.1 millimole per liter (mm/L), platelets 278 x 10^9^/L, sodium 141 mm/L, potassium 4.4 mm/L, glucose 94 milligram per decaliter (mg/dL), blood urea nitrogen 17 mg/dL, creatinine 0.9 mg/dL, calcium 9.3 mg/dL and normal liver function tests. A lipid panel showed low-density lipoproteins of 124 mg/dL, otherwise normal.

The patient was admitted to the stroke service for overnight observation. With a negative work-up and return to his baseline, his symptoms were ultimately attributed to facial baroparesis. The dysarthria, a poorly localized neurological deficit, was attributed to the facial muscle weakness. The patient was discharged with recommendations for nasal decongestant use prior to boarding future flights, as well as aspirin and a cholesterol-lowering agent, and follow-up for blood pressure monitoring.

## DISCUSSION

The pathophysiology behind facial baroparesis can be easily explained by the nerve’s anatomical course (Figure 1). The facial nerve exits the brainstem at the pontomedullary junction and traverses the cerebellopontine angle prior to entering the petrous portion of the temporal bone via the internal auditory meatus. It then travels through the facial canal, which is subdivided into three segments: the labyrinthine segment – giving off a branch to the greater petrosal nerve; the tympanic segment; and the mastoid segment – giving off branches to the stapedius and chorda tympani. The nerve then exits the skull via the stylomastoid foramen, traverses the parotid gland, and separates into five terminal branches that innervate the nerves of facial expression.^5^

The most widely accepted mechanism of facial nerve baroparesis is an ischemic neuropraxia occurring at the tympanic segment of the facial nerve. The tympanic portion of the facial canal traverses the middle ear cavity just medial to the incus. Here the facial nerve and middle ear are separated by only a thin layer of bone. In one study, spontaneous dehiscence of the tympanic portion of the canal was observed on CT in up to 55% of normal adults, resulting in direct communication between the facial nerve and the middle ear cavity.^6^

The middle ear is an enclosed, air-filled space. With an intact tympanic membrane, the only mechanism of pressure equalization is through the nasopharynx via the eustachian tube. With even a mild degree in eustachian tube dysfunction, it can be difficult to equalize the pressures in the middle ear with the outside environment. At a cruising altitude of 35,000 feet, the decrease in cabin pressure is estimated to be as high as 266 centimeters of water, a pressure that can easily overcome capillary hydrostatic pressure.^7^ It is thought that this increase in middle ear pressure is transmitted directly to the tympanic portion of the facial nerve, resulting in a temporary, ischemic neuropraxia. In our case, as in similar reported cases, all symptoms resolved shortly after equalization of middle ear and ambient pressures.^1^,^2^,^3^,^4^

**CPC-EM Capsule**

What do we already know about this clinical entity?

Facial baroparesis, first reported in divers, is a seventh cranial nerve palsy caused by transient hypoxemia of the facial nerve secondary to increased pressure in the middle ear cavity.

What makes this presentation of disease reportable?

*The incidence is unknown and only 23 cases have been reported in the available literature.8*

What is the major learning point?

Facial baroparesis is an under recognized condition potentially mimicking stroke, Bell’s Palsy, air embolism, or Type II decompression sickness.

How might this improve emergency medicine practice?

Entity awareness may result in less unnecessary testing, decreased long term ischemic nerve damage, and a reduction of inappropriate revocation of diving and/or aviation licenses.

## CONCLUSION

Facial baroparesis is thought to be caused by transient ischemia of seventh cranial nerve. Though uncommon, emergency physicians must consider this diagnosis when facial nerve complaints occur. Eliciting an accurate history, typically involving diving or flying, will lend itself to an accurate diagnosis. Symptoms should resolve upon equalization of middle ear and ambient pressures.

Documented patient informed consent and/or Institutional Review Board approval has been obtained and filed for publication of this case report.

## Figures and Tables

**Image:**
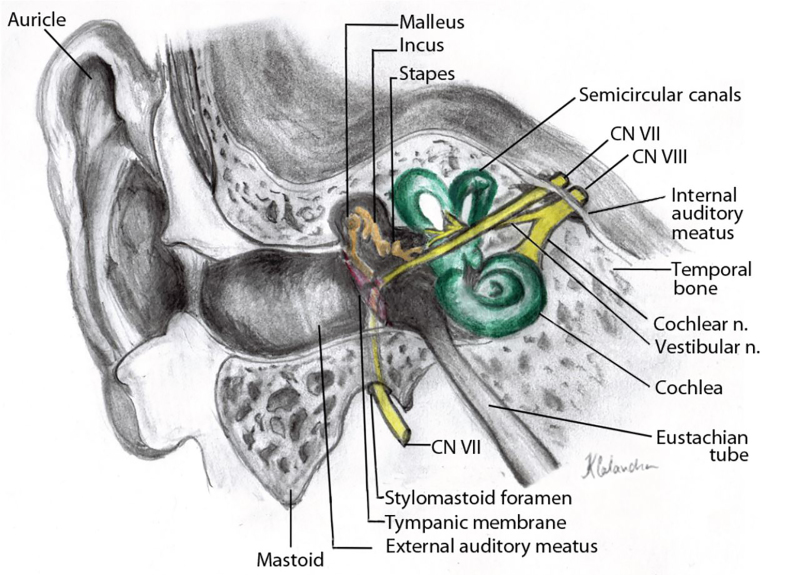
Anatomical course of the facial nerve through the facial canal, from entry via internal auditory meatus to exit at stylomastoid foramen. Illustrated by Kristin Calandra, PAC, MSPAS.
